# Network meta-analysis of efficacy and safety of drugs for the treatment of moderate to severe ulcerative colitis

**DOI:** 10.3389/fphar.2024.1481678

**Published:** 2025-01-03

**Authors:** Wenkai Zhang, Songbo Zhao, Jipin Li, Yihua Sun, Xiang Wang

**Affiliations:** ^1^ The Second Clinical College of Lanzhou University, Lanzhou, Gansu, China; ^2^ Department of Gastroenterology, Lanzhou University Second Hospital, Lanzhou, Gansu, China; ^3^ Department of General Surgery, Lanzhou University Second Hospital, Lanzhou, Gansu, China

**Keywords:** ulcerative colitis, drug treatment, efficacy, safety, network meta-analysis

## Abstract

**Purpose:**

To guide the drug selection for treatment of moderate to severe ulcerative colitis (UC) by evaluating the efficacy and safety of various drugs.

**Methods:**

This systematic review searched the Embase, PubMed, The Cochrane Library, and Web of Science databases and included randomized controlled trials (RCTs) based on the drugs used alone or in combination for treating UC. Moreover, the Stata17.0 software was employed for statistical analysis and results were reported as relative risk (RR) and 95% confidence interval (CI).

**Results:**

For the efficacy of induction, upadacitinib ranked first in clinical response, clinical remission, and endoscopic improvement rates, with cumulative probabilities of 96.0%, 99.3%, and 99.0%, respectively. Moreover, for the efficacy of maintenance, upadacitinib ranked first in both clinical remission and endoscopic improvement with a cumulative probability of 93.2% and 93.3%, respectively. For safety, vedolizumab showed the best incidence of adverse events (AE) with 16.8% cumulative probability, while upadacitinib showed the best incidence of serious adverse events (SAE) with 13.8% cumulative probability.

**Conclusion:**

In a systematic review and network meta-analysis, we found upadacitinib showed the best efficacy and safety in to be ranked highest in patients with moderate to severe ulcerative colitis. More trials of direct comparisons are needed to inform clinical decision making with greater confidence.

## Introduction

Inflammatory bowel disease is a group of chronic non-specific intestinal inflammatory diseases. UC is one such disease with a partly understood pathogenesis. Reportedly, genetic susceptibility, immunomodulatory dysfunction, microbiota, environment, and other factors contribute to intestinal inflammatory response. UC is characterized by continuous and superficial mucosal inflammation which extends to the proximal colon, leading to ulcers, massive bleeding, toxic megacolon, and fulminant colitis when the lesions are severe (Chang, 2020). The increasing number of UC patients worldwide has rendered it a common disease of the digestive system (Ng et al., 2017). The disease and its related complications (infection, thrombosis, malignant tumors, etc.) impose a huge medical and economic burden on the patient’s family and society (Alatab et al., 2020).

The main objective of UC treatment is to achieve clinical remission and mucosal healing (Cazzato et al., 2021). At present, commonly used drugs, such as 5-aminosalicylic acid (5-ASA), hormones, and immunosuppressants, can relieve the symptoms of patients without terminating the mucosal inflammatory activity and disease development. Moreover, severe short-term (systemic immunosuppression and risk of opportunistic infections) and long-term (Cushing’s syndrome, diabetes, and osteoporosis) adverse effects limit their application (Na and Moon, 2019). Over the past 2 decades, the emergence of biological agents (i.e. anti-tumor necrosis factor (TNF), anti-α4β7 integrin, anti-IL12/23) and small molecule drugs (SMDs) (i.e. Janus kinase (JAK) inhibitors, sphingosine 1-phosphate (S1P) receptor modulators), has shown potential of inducing and maintaining the clinical remission and mucosal healing of UC (Pouillon et al., 2016; Kim and Cheon, 2017).

Consequently, the treatment of moderate to severe UC has shown promising results. Various randomized controlled trials (RCTs) studies have confirmed the efficacy and safety of these drugs (Suzuki et al., 2015; Suzuki et al., 2014; Sands et al., 2019a; Sandborn et al., 2012; Sandborn et al., 2017; Sandborn et al., 2014; Sandborn et al., 2020; Sandborn et al., 2021; Danese et al., 2022); however, comparative studies of these drugs are limiting. In an ideal world physicians would like to select the most efficacious and least toxic drug upfront. While conventional meta-analysis has a limited scope, network meta-analysis performs direct and indirect comparisons of evidence to rank and compare the efficacy and safety of multiple interventions and to select an optimal intervention. Therefore, we conducted a network meta-analysis to compare the efficacy and safety of different drugs, providing a basis for selecting clinical treatments for moderate to severe UC.

## Methods

### Data source and search strategy

This systematic review searched the Embase, PubMed, the Cochrane Library, and Web of Science databases from inception to 31 March 2023, to collect literature related to drug treatment of UC, using a predefined search strategy. The retrieval strategy was standardized following several pre-searches and manual cross-referencing of the included articles in the English language only.

### Inclusion and exclusion criteria

Inclusion criteria were as follows: (1) Subjects: patients with a definite diagnosis of UC following the diagnostic criteria of the American Gastroenterological Association (Rubin et al., 2019). The Mayo (Schroeder et al., 1987) or Adapted Mayo (Danese et al., 2022) scores (excluding physician’s evaluation) were 6–12 and 5–9 points, respectively, whereas the endoscopic subscore was 2–3 points. Age ≥15 years old, irrespective of gender or race; (2) Interventions: the experimental group was treated with hormones, ASA, immunosuppressive or biological agents, and SMDs (alone or in combination), whereas the control group was treated with different drugs or placebo. The medications approved by the United States Food and Drug Administration (USFDA) for the treatment of moderate to severe UC (infliximab, adalimumab, vedolizumab, ustekinumab, golimumab, tofacitinib, upadacitinib, and ozanimod) were examined. The dose and method of the intervention were the same while including only Phase III clinical trials. All the treatments were continued for ≥2 weeks; (3) Outcomes: Clinical response, clinical remission, and endoscopic improvement rates in the induction period. Clinical remission and endoscopic improvement rates during the maintenance period. Safety outcomes included the incidence of AE and SAE. Clinical response (D'Haens et al., 2007) indicated a decrease in the Mayo score by ≥ 30% and ≥3 points from the baseline (or Adapted Mayo score by ≥ 2 points from the baseline), with a decrease in the rectal bleeding component (≥1 point) or subscore (0 or 1) of the Mayo scale. Clinical remission (D'Haens et al., 2007) was defined as a total Mayo or Adapted Mayo score of ≤2 and none of the subscores >1. Endoscopy improvement (D'Haens et al., 2007) indicated a Mayo endoscopic subscore of 0 or 1.

Observational, cohort, case-control studies, and case reports were excluded. In addition, for patients with mild to moderate UC, studies lacking reports on drug efficacy or safety, open-label, or non-English RCTs were excluded. Moreover, studies without full-text access were excluded.

### Literature screening and data extraction

The retrieved data was imported into Endnote X9 to search and remove the duplicate literature. Two researchers independently screened the literature according to the inclusion and exclusion criteria. The initial screening was based on the title and abstract, whereas the final screening for literature inclusion was based on full-text reading. The baseline characteristics, study design, interventions, outcomes, and risk of bias were recorded on a Microsoft Excel spreadsheet. A third reviewer resolved disagreements or conflicts, if any. If the doses were inconsistent, we selected the FDA-approved dose. The following points were considered during data extraction (Cholapranee et al., 2017; Wheat et al., 2017; Singh et al., 2020a): (1) Follow-up time of ≤24 weeks was included in the induction trials, and >24 weeks was included in the maintenance trials. If a study provided different endpoint periods (e.g. at week 30 and week 52), we considered the longer time. (2) Owing to the lack of re-randomization of infliximab, infliximab/AZA, adalimumab, and vedolizumab (not including VARSITY 2019 (Sands et al., 2019b)) at the end of the induction period, the outcome of clinical response in maintenance was not included. (3) Safety is less susceptible to experimental design. A longer follow-up time indicates a more accurate incidence of AE and SAE. Finally, maintenance endpoints were extracted, if reported for both the induction and maintenance.

### Quality assessment

Risk-of-bias assessment was performed independently by two authors using the Cochrane risk-of-bias tool to classify each study as having a low, medium, or high risk of bias. Seven domains were assessed using this tool, including incomplete outcome data, selective outcome reporting, allocation concealment, blinding of participants and personnel, random sequence generation, blinding of outcome assessment, and other potential sources of bias. The risk of bias was plotted by Review Manager 5.4.1 software.

### Statistical analysis

The results were reported as relative risk (RR) and 95% confidence interval (CI). Heterogeneity test and direct meta-analysis were performed by Stata17.0 software. Network meta-analysis to draw the evidence network diagram employed the traditional frequency method. The effectiveness and safety of interventions were ranked by surface under the cumulative ranking (SUCRA) ranging from 0 to 1. The comparison-corrected funnel plot was used to evaluate the small sample effect and publication bias.

## Results

### Search results and study characteristics

**FIGURE 1:**
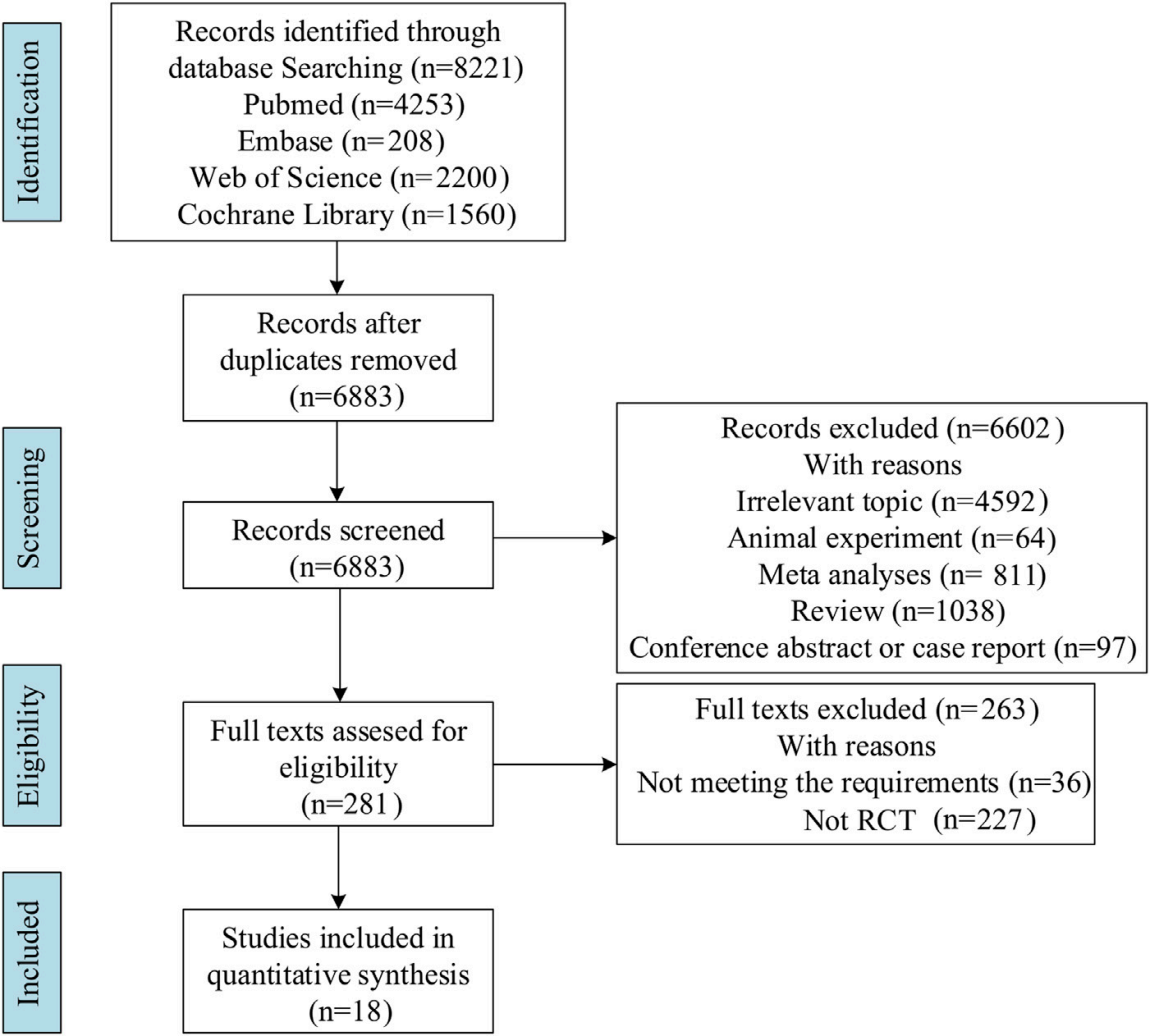
Flow diagram of evidence search and selection process.

Literature search identified 8,221 relevant articles in PubMed, Embase, The Cochrane Library, and Web of Science. Based on the title, abstract, and full text, 18 eligible RCTs were included (Figure 1). A total of 18 articles (22 RCTs) were included, involving 7,873 patients with moderate to severe UC, and divided into the experimental (4,621) and control (3,252) groups. All the studies, including 18 induction and 14 maintenance RCTs were randomized, double-blind, and controlled drug clinical trials. In addition, the basic characteristics, such as gender, age, and Mayo score [except for one literature (Jiang et al., 2015)], were balanced and comparable (Table 1). The treatment regimens (dose, route of administration, and duration of treatment), duration of follow-up, and outcomes during the induction and maintenance periods are shown in Tables 2, 3. Among them, the sample of tacrolimus was too small to be included in the network meta-analysis.

**TABLE 1:** Characteristics of the included trials.

Study	Nation	Number of site	Treatment				Comparator			
			Number of people (F/M)	Age (yr)	Mayo score	Intervention	Number of people (F/M)	Age (yr)	Mayo score	Intervention
ACT 1 2005^c^	Belgium	62	121 (78/43)	42.4 ± 14.3	8.5 ± 1.7	Infliximab	121 (72/49)	41.4 ± 13.7	8.4 ± 1.8	Placebo
ACT 2 2005^a^	Belgium	55	121 (76/45)	40.5 ± 13.1	8.3 ± 1.5	Infliximab	123 (71/52)	39.3 ± 13.5	8.5 ± 1.5	Placebo
Jiang 2015^c^	China	1	41 (26/15)	34.3 ± 14.3	NR	Infliximab	41 (25/16)	34.5 ± 14.9	NR	Placebo
Panaccione 2014^a^	Canada	NR	78 (42/36)	38.5 ± 12.7	8.1 ± 1.4	Infliximab	80 (48/32)	38.0 ± 12.2	8.6 ± 1.3	Infliximab/AZA
Reinisch 2011^a^	Austria	94	130 (83/47)	36.5 (18–75)	8.8 ± 1.61	Adalimumab	130 (82/48)	37 (18–72)	8.7 ± 1.56	Placebo
Sandborn 2012^c^	America	103	248 (142/106)	39.6 ± 12.47	8.9 ± 1.50	Adalimumab	246 (152/94)	41.3 ± 13.22	8.9 ± 1.75	Placebo
Suzuki 2014^c^	Japan	65	90 (61/29)	42.5 ± 14.6	8.6 ± 1.4	Adalimumab	96 (70/26)	41.3 ± 13.6	8.5 ± 1.6	Placebo
VARSITY 2019^c^	America	245	386 (216/170)	40.5 ± 13.4	8.7 ± 1.5	Adalimumab	385 (234/151)	40.8 ± 13.7	8.7 ± 1.6	Vedolizumab
Sandborn 2019^b^	America	141	54 (31/23)	41.6 ± 14.1	9.0 (6–12)	Vedolizumab	56 (34/22)	39.4 ± 11.7	9.0 (6–11)	Placebo
Feagan 2014^c^	Canada	211	225 (132/93)	40.1 ± 13.1	8.5 ± 1.8	Vedolizumab	149 (92/57)	41.2 ± 12.5	8.6 ± 1.7	Placebo
Motoya 2019^c^	Japan	100	164 (99/65)	42.3 ± 14.4	8.3 ± 1.5	Vedolizumab	82 (55/27)	44.0 ± 16.0	8.1 ± 1.5	Placebo
Sands 2019^c^	America	244	322 (195/127)	41.7 ± 13.7	8.9 ± 1.5	Ustekinumab	319 (197/122)	41.2 ± 13.5	8.9 ± 1.6	Placebo
Sandborn 2014^c^	America	217	258 (140/118)	39.7 ± 13.79	8.7 ± 1.60	Golimumab	258 (130/128)	39.7 ± 13.35	8.3 ± 1.52	Placebo
Hibi 2017^b^	Japan	49	32 (19/13)	39.30 ± 12.00	8.0 (6; 11)	Golimumab	31 (19/12)	42.90 ± 14.41	8.0 (6; 12)	Placebo
OCTAVE 1 2017^a^	America	144	476 (277/199)	41.3 ± 14.1	9.0 ± 1.4	Tofacitinib	122 (77/45)	41.8 ± 15.3	9.1 ± 1.4	Placebo
OCTAVE 2 2017^a^	America	169	429 (259/170)	41.1 ± 13.5	9.0 ± 1.5	Tofacitinib	112 (55/57)	40.4 ± 13.2	8.9 ± 1.5	Placebo
OCTAVE S 2017^b^	America	297	198 (103/95)	41.9 ± 13.7	3.3 ± 1.8	Tofacitinib	198 (116/82)	43.4 ± 14.0	3.3 ± 1.8	Placebo
UC 1 2022^a^	Italy	199	319 (198/121)	43.0 ± 23.0	7.0 ± 1.2	Upadacitinib	154 (97/57)	44.5 ± 23.0	7.0 ± 1.2	Placebo
UC 2 2022^a^	Italy	204	341 (214/127)	40.0 ± 24.0	7.0 ± 1.2	Upadacitinib	174 (107/67)	42.0 ± 24.0	7.0 ± 1.2	Placebo
UC 3 2022^b^	Italy	195	148 (95/53)	40.0 ± 22.0	7.0 ± 1.2	Upadacitinib	149 (85/64)	40.0 ± 21.0	7.0 ± 1.2	Placebo
Sandborn 2021^c^	America	285	429 (245/184)	41.4 ± 13.5	8.9 ± 1.5	Ozanimod	216 (143/73)	41.9 ± 13.6	8.9 ± 1.4	Placebo
Lawrance 2017^a^	Australia	4	11 (8/3)	48.4 ± 4.9	8.6 ± 0.4	Tacrolimus	10 (4/6)	39.0 ± 4.8	9.6 ± 0.5	Placebo

^a^ induction period.

^b^ maintenance period.

^c^ induction period and maintenance period.

**TABLE 2:** Intervention parameters, duration of follow-up, and outcomes of the induction trials.

Study	Intervention parameters		Follow-up duration	Outcomes
	Treatment	Comparator		
ACT 1 2005	Infliximab 5 mg/Kg IV W0, 2, 6	Placebo	8W	➀➁➂
ACT 2 2005	Infliximab 5 mg/Kg IV W0, 2, 6	Placebo	8W	➀➁➂
Jiang 2015	Infliximab 5 mg/Kg IV W0, 2, 6	Placebo	8W	➀➁➂
Panaccione 2014	Infliximab 5 mg/Kg IV W0, 2, 6, 14+ Placebo PO	Infliximab 5 mg/Kg IV W0, 2, 6, 14+AZA PO 2.5 mg/kg/d	16W	➀➂➃➄
Reinisch 2011	Adalimumab SC W0:160 mg W2:80 mg W4:40 mg W6:40 mg	Placebo	8W	➀➁➂➃➄
Sandborn 2012	Adalimumab SC W0:160 mg W2:80 mg W4:40 mg W6:40 mg	Placebo	8W	➀➁➂
Suzuki 2014	Adalimumab SC W0:160 mg W2:80 mg W4:40 mg W6:40 mg	Placebo	8W	➀➁➂➃➄
VARSITY 2019	Adalimumab SC W0:160 mg W2:80 mg W4:40 mg W6:40 mg + Placebo IV	Vedolizumab 300 mg IV W0, 2, 6+ Placebo SC	14W	➀➁
Feagan 2014	Vedolizumab 300 mg IV W0, 2	Placebo	6W	➀➁➂
Motoya 2019	Vedolizumab 300 mg IV W0, 2, 6	Placebo	10W	➀➁➂➃➄
Sands 2019	Ustekinumab 6 mg/kg IV W0	Placebo	8W	➀➁➂➃➄
Sandborn 2014	Golimumab SC W0:200 mg W2:100 mg	Placebo	6W	➀➁➂➃➄
OCTAVE 1 2017	Tofacitinib 10 mg PO Bid	Placebo	8W	➀➁➂➃➄
OCTAVE 2 2017	Tofacitinib 10 mg PO Bid	Placebo	8W	➀➁➂➃➄
UC 1 2022	Upadacitinib 45 mg PO Qd	Placebo	8W	➀➁➂➃➄
UC 2 2022	Upadacitinib 45 mg PO Qd	Placebo	8W	➀➁➂➃➄
Sandborn 2021	Ozanimod 0.92 mg PO Qd	Placebo	10W	➀➁➂➃➄
Lawrance 2017	Tacrolimus 0.5 mg/mL 3 mL PR Bid	Placebo	8W	➀➁➂

IV, intravenous; SC, subcutaneous injection; PO, oral; PR, intrarectal medication; Bid, twice a day; Qd, once a day; QOW, every 1 week; Q4W, every 4 weeks; Q8W, every 8 weeks; ➀, clinical response; ➁, clinical remission; ➂, endoscopic improvement; ➃, AE; ➄, SAE.

**TABLE 3:** Intervention parameters, duration of follow-up, and outcomes of the maintenance trials.

Study	Intervention parameters		Follow-up duration	Outcomes
	Treatment	Comparator		
ACT 1 2005	Infliximab 5 mg/Kg IV W14, 22, 30, 38, 46	Placebo	54W	➀➁➂➃➄
Jiang 2015	Infliximab 5 mg/Kg IV W14, 22	Placebo	30W	➀➁➂➃➄
Sandborn 2012	Adalimumab 40 mg SC QOW	Placebo	52W	➀➁➂➃➄
Suzuki 2014	Adalimumab 40 mg SC QOW	Placebo	52W	➁➂
VARSITY 2019	Adalimumab 40 mg SC QOW + Placebo IV	Vedolizumab 300 mg IV W14, 22, 30, 38, 46+ Placebo SC	52W	➁➂➃➄
Sandborn 2019	Vedolizumab 300 mg IV Q8W	Placebo	6–52W	➁➂➃➄
Feagan 2014	Vedolizumab 300 mg IV Q8W	Placebo	52W	➁➂➄
Motoya 2019	Vedolizumab 300 mg IV Q8W	Placebo	60W	➁➂➃➄
Sands 2019	Ustekinumab 90 mg IV Q8W	Placebo	52W	➁➂➃➄
Sandborn 2014	Golimumab 100 mg SC Q4W	Placebo	54W	➀➁➂➃➄
Hibi 2017	Golimumab 100 mg SC Q4W	Placebo	6–54W	➀➂➃➄
OCTAVE S 2017	Tofacitinib 5 mg PO Bid	Placebo	52W	➀➁➂➃➄
UC 3 2022	Upadacitinib 15 mg PO Qd	Placebo	52W	➀➁➂➃➄
Sandborn 2021	Ozanimod 0.92 mg PO Qd	Placebo	52W	➀➁➂➃➄

IV, intravenous; SC, subcutaneous injection; PO, oral; Bid, twice a day; Qd, once a day; QOW, every 1 week; Q4W, every 4 weeks; Q8W, every 8 weeks; ➀, clinical response; ➁, clinical remission; ➂, endoscopic improvement; ➃, AE; ➄, SAE.

### Quality assessment

**FIGURE 2:**
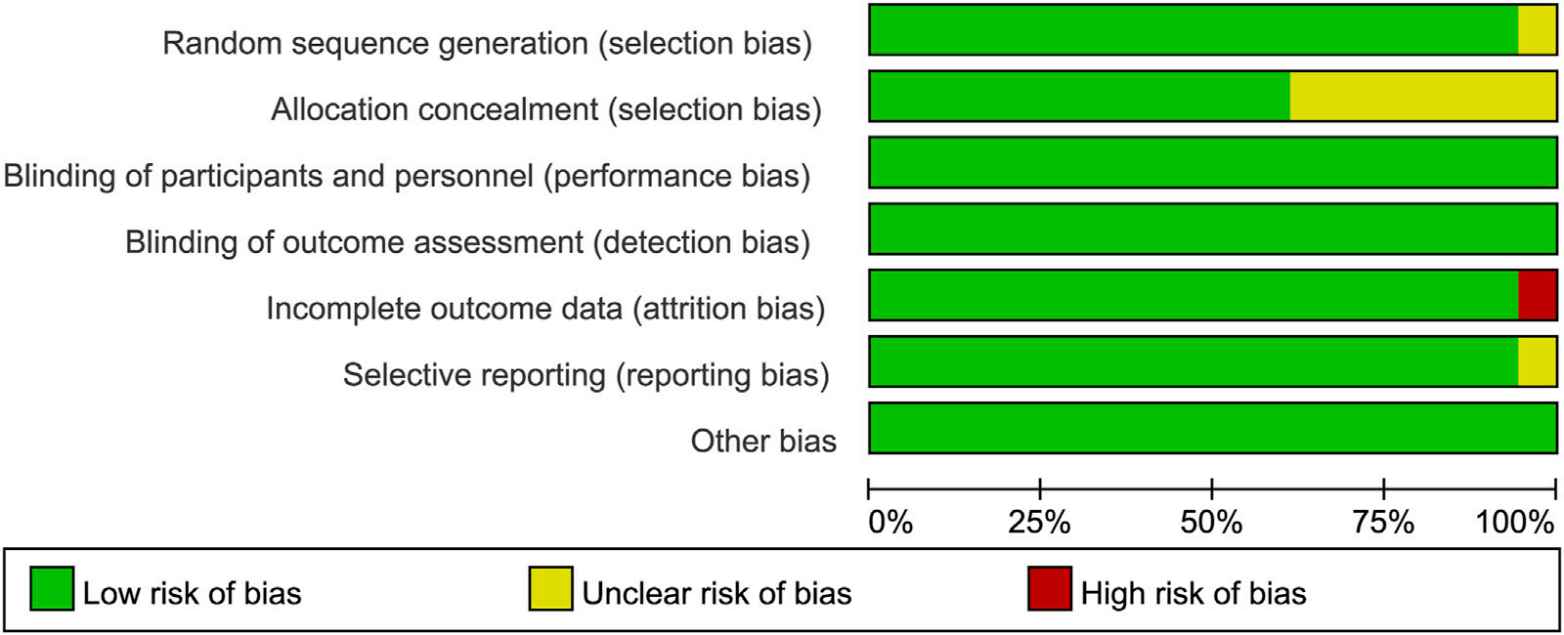
Quality assessment diagram.

All the trials were double-blind and bias-free. While one study did not mention the random sequence generation method, eight studies did not clarify whether the allocation was concealed. Nonetheless, one study was incompletely reported, whereas the risk of selective reporting of another study was unclear. The bar chart of quality assessment results was drawn by RevMan 5.4.1 software (Figure 2).

### Direct treatment comparisons

#### Induction of clinical response, clinical remission, and endoscopic improvement

**FIGURE 3:**
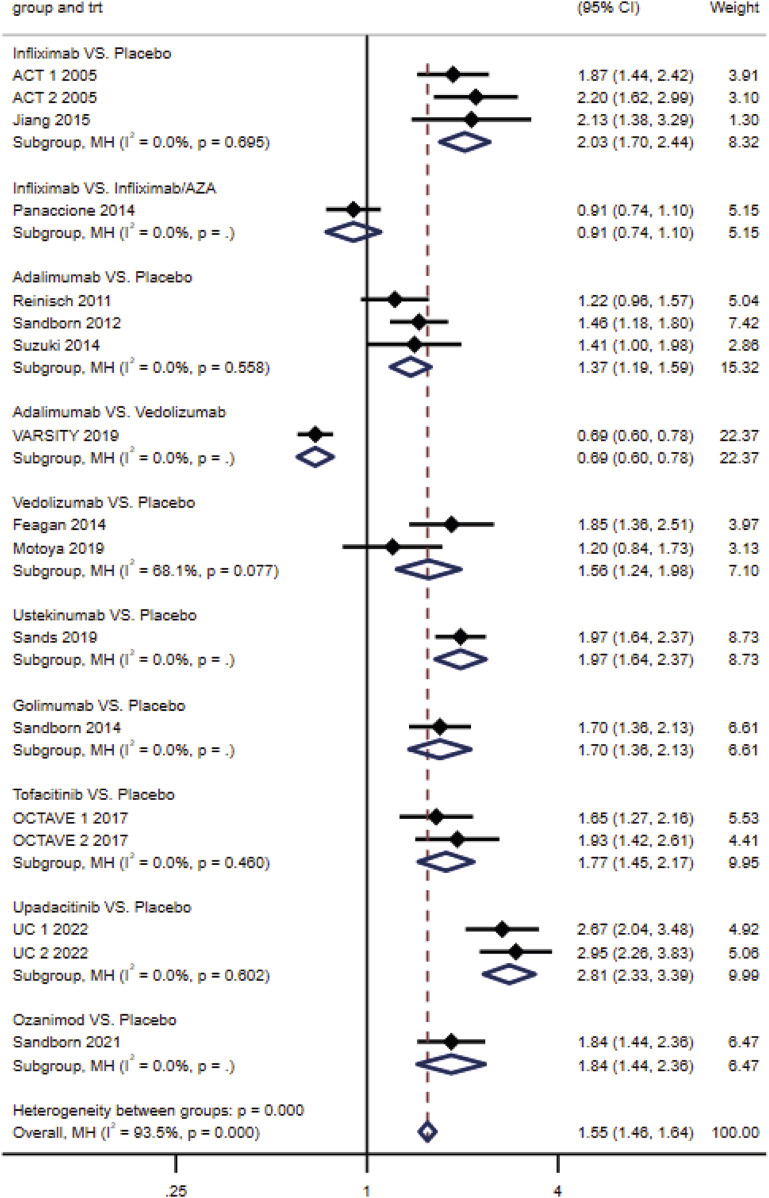
Effectiveness of drugs in ulcerative colitis compared to placebo: forest plot of direct meta-analysis of clinical response rate in induction phase.

No heterogeneity across studies of the same intervention (*p* ≥ 0.10) was reported. The results of direct meta-analysis showed that all interventions were more effective than placebo for three endpoints. The clinical response rate of vedolizumab was higher than that of adalimumab (RR, 0.69; 95% CI, 0.60–0.78) (Figure 3); however, the difference between their clinical remission rates was not statistically significant. Similarly, no significant difference was observed between infliximab and infliximab/AZA in inducing clinical response and endoscopic improvement (Figure 3; Supplementary Figures S1A, B).

#### Maintenance of clinical remission and endoscopic improvement

No heterogeneity was observed across studies of the same intervention (*p* ≥ 0.10). Direct comparisons showed that all interventions were more effective than placebo for the maintenance of clinical remission and endoscopic improvement. Compared with vedolizumab, adalimumab has lower clinical response rate (RR, 0.72; 95% CI, 0.57–0.92) and endoscopic improvement rate (RR, 0.70; 95% CI, 0.57–0.86) (Supplementary Figures S1C, D).

#### Safety of treatments

There was no heterogeneity across studies of the same intervention (*p* ≥ 0.10). The results of direct meta-analysis revealed no significant difference in the incidence of AE between infliximab, adalimumab, vedolizumab, ustekinumab, tofacitinib, and upadacitinib relative to placebo. Additionally, no significant difference in AE rates was observed between infliximab and infliximab/AZA, unlike that between vedolizumab and adalimumab. Notably, the incidence of AE in golimumab (RR, 1.16; 95% CI, 1.02–1.31) and ozanimod (RR, 1.34; 95% CI, 1.08–1.67) was significantly higher than that in placebo. Moreover, the results of the direct meta-analysis showed no significant difference in the incidence of SAE among all interventions (Supplementary Figures S1E, F).

### Network meta-analysis

#### Induction of clinical response

The clinical response rates of infliximab, infliximab/AZA, adalimumab, vedolizumab, ustekinumab, golimumab, tofacitinib, upadacitinib, and ozanimod were superior to those of placebo. However, infliximab, infliximab/AZA, vedolizumab, ustekinumab, tofacitinib, and upadacitinib showed higher rates than adalimumab. Specifically, the clinical response rate of upadacitinib was superior to that of vedolizumab, ustekinumab, tofacitinib, and ozanimod (Table 4). Moreover, the SUCRA probability ranking was as follows: upadacitinib (96.0), infliximab/AZA (86.4), infliximab (75.0), ustekinumab (63.4), tofacitinib (45.6), ozanimod (43.3), vedolizumab (40.6), golimumab (37.3), adalimumab (12.4), and placebo (0.1) (Supplementary Figure S2A).

**TABLE 4:** Network meta-analysis of clinical response rate during induction.

Infliximab									
0.71 (0.32,1.57)	Infliximab/AZA								
**2.96 (1.74,5.03)**	**4.18 (1.60,10.93)**	Adalimumab							
1.72 (0.97,3.06)	2.43 (0.91,6.53)	**0.58 (0.39,0.86)**	Vedolizumab						
1.24 (0.63,2.42)	1.75 (0.62,4.98)	**0.42 (0.23,0.76)**	0.72 (0.38,1.36)	Ustekinumab					
1.83 (0.92,3.65)	2.59 (0.90,7.45)	0.62 (0.33,1.15)	1.07 (0.55,2.05)	1.48 (0.70,3.11)	Golimumab				
1.62 (0.89,2.95)	2.29 (0.84,6.22)	**0.55 (0.33,0.92)**	0.94 (0.54,1.65)	1.31 (0.67,2.53)	0.88 (0.45,1.74)	Tofacitinib			
0.56 (0.31,1.02)	0.79 (0.29,2.15)	**0.19 (0.11,0.32)**	**0.33 (0.19,0.57)**	**0.45 (0.23,0.87)**	**0.31 (0.16,0.60)**	**0.35 (0.19,0.62)**	Upadacitinib		
1.68 (0.84,3.34)	2.37 (0.83,6.82)	0.57 (0.31,1.05)	0.98 (0.51,1.88)	1.36 (0.65,2.84)	0.92 (0.43,1.95)	1.04 (0.53,2.04)	**3.00 (1.53,5.89)**	Ozanimod	
**4.39 (2.85,6.75)**	**6.21 (2.50,15.41)**	**1.48 (1.09,2.03)**	**2.55 (1.75,3.71)**	**3.54 (2.12,5.92)**	**2.39 (1.40,4.10)**	**2.71 (1.79,4.11)**	**7.84 (5.20,11.84)**	**2.61 (1.53,4.46)**	Placebo

Comparisons should be read from left to right. Numbers in bold are statistically significant. Numbers in parentheses indicate 95% CI.

#### Induction of clinical remission

Results revealed that infliximab, adalimumab, vedolizumab, ustekinumab, golimumab, tofacitinib, upadacitinib, and ozanimod had better clinical remission rates than placebo. Within the treatment group, infliximab showed a superior clinical remission rate than adalimumab and vedolizumab. Similarly, upadacitinib was superior to that of adalimumab, vedolizumab, ustekinumab, golimumab, tofacitinib, and ozanimod (Supplementary Table S1A). Moreover, the SUCRA probability ranking was as follows: upadacitinib (99.3), infliximab (79.3), ozanimod (60.3), ustekinumab (55.2), tofacitinib (54.4), golimumab (53.7), vedolizumab (33.2), adalimumab (14.5), and placebo (0) (Supplementary Figure S2B).

#### Induction of endoscopic improvement

As expected, infliximab, infliximab/AZA, adalimumab, vedolizumab, ustekinumab, golimumab, tofacitinib, upadacitinib, and ozanimod had superior endoscopic improvement rates than placebo. Additionally, infliximab and infliximab/AZA were superior to adalimumab, vedolizumab, and golimumab, whereas tofacitinib showed a higher endoscopic improvement rate than adalimumab. Similarly, upadacitinib was superior to infliximab, adalimumab, vedolizumab, ustekinumab, golimumab, tofacitinib, and ozanimod (Supplementary Table S1B). Moreover, the SUCRA probability ranking was as follows: upadacitinib (99.0), infliximab/AZA (84.4), infliximab (73.4), ozanimod (62.5), tofacitinib (58.6), ustekinumab (47.8), golimumab (30.7), vedolizumab (27.3), adalimumab (16.3), and placebo (0) (Supplementary Fgiure S2C).

#### Maintenance of clinical remission

The clinical remission rates of infliximab, adalimumab, vedolizumab, ustekinumab, golimumab, tofacitinib, upadacitinib, and ozanimod were higher than that of placebo. Vedolizumab had a superior clinical remission rate than adalimumab. The clinical remission rate of upadacitinib was superior to that of adalimumab, ustekinumab, golimumab, and ozanimod (Supplementary Table S1C). The SUCRA probability ranking was as follows: upadacitinib (93.2), tofacitinib (80.6), vedolizumab (77.2), infliximab (50.7), ozanimod (41.8), adalimumab (38.5), ustekinumab (38.1), golimumab (29.8), and placebo (0) (Supplementary Figure S2D).

#### Maintenance of endoscopic improvement

Infliximab, adalimumab, vedolizumab, ustekinumab, golimumab, tofacitinib, upadacitinib, and ozanimod had superior endoscopic improvement rates than placebo. Within the treatment group, infliximab, vedolizumab, tofacitinib, and upadacitinib showed superior endoscopic improvement rates than adalimumab. Similarly, upadacitinib was superior to ustekinumab, golimumab, and ozanimod (Supplementary Table S1D). Moreover, the SUCRA probability ranking was as follows: upadacitinib (93.3), tofacitinib (75.1), infliximab (73.7), vedolizumab (72.4), ustekinumab (41.8), golimumab (39.2), ozanimod (32.2), adalimumab (22.4), and placebo (0) (Supplementary Figure S2E).

#### Safety

The incidence of AE was lower for vedolizumab than golimumab; however, adverse effects were more common with ozanimod compared with adalimumab, vedolizumab, tofacitinib, and placebo (Supplementary Table S1E). Moreover, the SUCRA probability ranking of AE was as follows: ozanimod (87.6), golimumab (82.1), infliximab/AZA (68.5), infliximab (55.3), upadacitinib (50.8), adalimumab (46.6), placebo (38.8), ustekinumab (30.3), tofacitinib (23.1), and vedolizumab (16.8) (Supplementary Table S2F). In contrast, the incidence of SAE was not statistically significant for interventions (Supplementary Table S1F), and the SUCRA probability ranking of SAE was as follows: infliximab/AZA (88.2), golimumab (85.6), placebo (65.3), adalimumab (52.8), ustekinumab (48.3), infliximab (40.0), vedolizumab (39.6), tofacitinib (39.1), ozanimod (27.3), and upadacitinib (13.8) (Supplementary Figure S2G).

### Network diagram and detection of inconsistency

**FIGURE 4:**
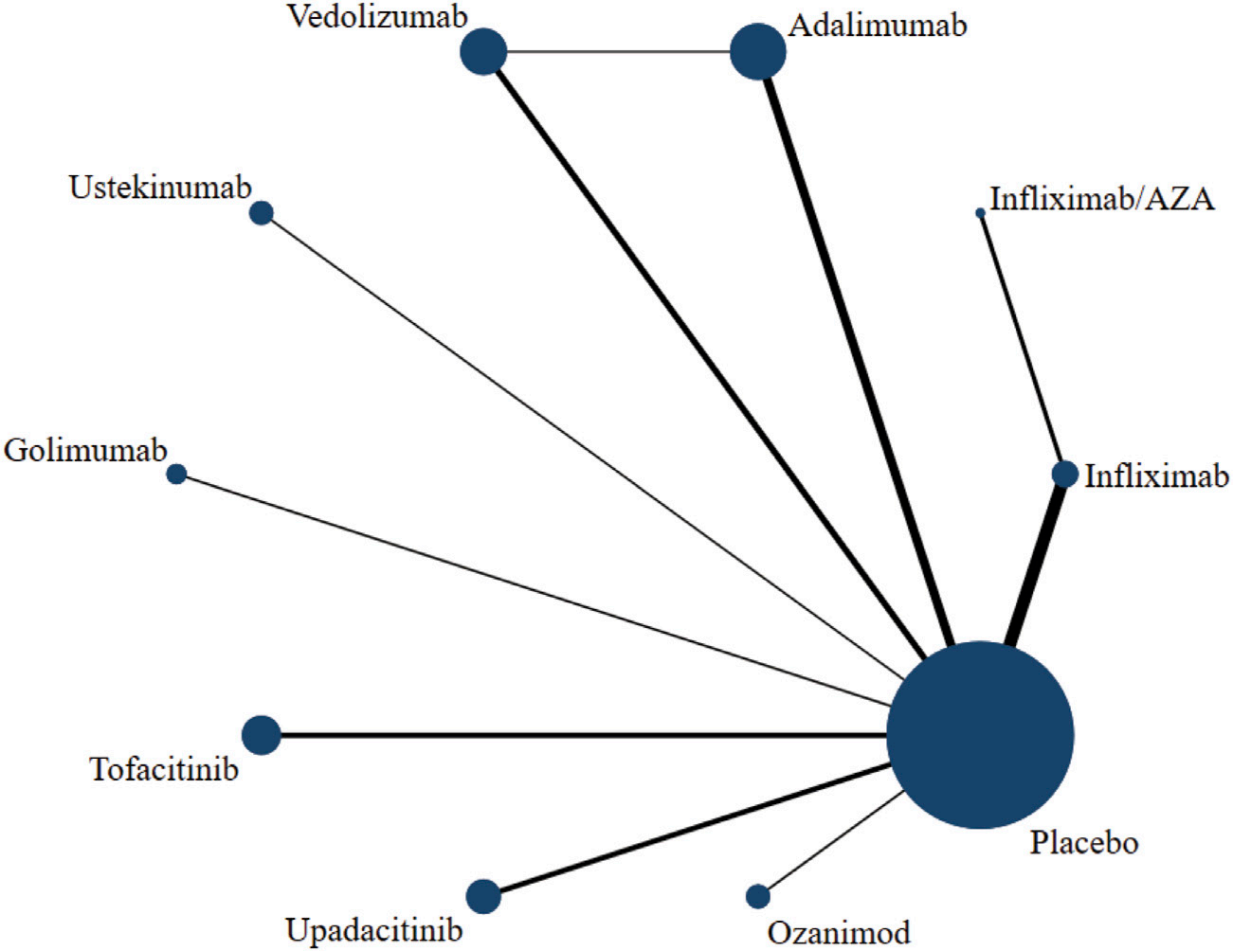
Network diagram of clinical response during induction.

The network diagram presented the intervention measures of the relationship, where dots and lines represented interventions and direct comparisons, respectively. Similarly, dot size and line thickness indicated sample size and quantity, respectively. The network diagram provided an intuitive view of potential direct comparisons between interventions (Figure 4; Supplementary Figures S3A–F). A closed loop of adalimumab-vedolizumab-placebo was formed among the six outcome indicators: induction of clinical response, induction of clinical remission, maintenance of clinical remission, maintenance of endoscopic improvement, incidence of AE, and incidence of SAE. Results showed inconsistent clinical response rates during the induction period (*p* = 0.024); however, the rest of the outcome indicators showed a good consistency (*p* > 0.05).

### Publication bias or small sample effect

Supplementary Fgiures S4A–G showed the “comparison-corrected” funnel plots of the induction clinical response, induction clinical remission, induction endoscopic improvement, maintenance clinical remission, maintenance endoscopic improvement, AE, and SAE, respectively. No significant asymmetry among the points in the funnel plots of Supplementary Figures 4A–D, 4G was observed, indicating a lack of publication bias. In contrast, funnel plots in Supplementary Fgiures S4E, F showed partial symmetry. Further analysis revealed that Hibi 2017 (Hibi et al., 2017) (golimumab vs. placebo) had a small sample effect, while the other points had no obvious asymmetry, indicating less possibility of publication bias.

### Subgroup analysis

Two different study designs were analyzed during the maintenance phase. One of these included vedolizumab, ustekinumab, golimumab, tofacitinib, upadacitinib, and ozanimod randomized patients, who achieved clinical responses during induction. However, studies of infliximab, infliximab/AZA, adalimumab, and vedolizumab (excluding VARSITY 2019 (Sands et al., 2019b)) did not re-randomize patients at the end of the induction period. To study the effect of different study designs on ranking results during the maintenance period, we performed subgroup analyses during the maintenance period to obtain the following results: (1) Clinical remission rate showed that the top three drugs remained upadacitinib, tofacitinib, and vedolizumab. (2) The endoscopic improvement rate showed that the probability ranking did not change significantly. See Supplementary Fgiures S5A–H for the network diagram and the probability ranking diagram.

## Discussion

In this updated systematic review and network meta analysis combining direct and indirect evidence from the RCTs of biologics (infliximab, adalimumab, vedolizumab, ustekinumab, and golimumab), SMDs (tofacitinib, upadacitinib, and ozanimod), and immunosuppressors (AZA and tacrolimus), we made several key observations. First, although all approved agents are effective, upadacitinib was ranked highest in terms of induction and maintenance. Second, the safety of vedolizumab is unequivocal compared to other active interventions, as it has the lowest rate of AE, followed by upadacitinib. Of note, upadacitinib had the lowest incidence of SAE. Third, when compared to infliximab alone, infliximab combined with AZA can enhance efficacy while potentially increasing the incidence of adverse events, particularly serious adverse events. As compared with previous estimates, this study expanded the scope of the search to include traditional drugs, biologics, and small molecule drugs. Although only two studies, Lawrance et al. (2017) and Panaccione et al. (2014), had conventional drugs that met the criteria, Lawrance et al. (2017) had to be excluded from the mesh analysis due to its small sample size. This study included high-quality RCTs characterized by low heterogeneity and a rigorous methodology. The updated analysis has key strengths with inclusion of a head-to-head trial comparing vedolizumab and adalimumab (Sands et al., 2019b), which forms a more connected network, and provides more robust. Additionally, it analyzed both the induction and maintenance phases, conducting a subgroup analysis (treat straight-through vs rerandomization of responders) on the outcome indicators during the maintenance phase, which enhances the overall persuasiveness of the results. With limited head-to-head trials, this information can inform clinical practice and guidelines directly and facilitate shared decision making for management of patients with moderate–severe ulcerative colitis.

Our results confirm several prior observational comparative effectiveness studies, individual patient level analyses of clinical trials, and indirect treatment comparison network meta-analyses suggesting higher efficacy and effectiveness of infliximab over adalimumab and golimumab (Bonovas et al., 2018; Singh et al., 2018; Singh et al., 2017; Singh et al., 2020b). This may be related to differences in pharmacokinetics and bioavailability with different dosing schema (weight-based vs fixed dose) and route of administration. Infliximab binds to TNF-α at the site of inflammation by intravenous administration, thereby inhibiting inflammation with maximum bioavailability. Therefore, it can induce immediate clinical responses in patients with moderate to severe UC. After subcutaneous administration, adalimumab and golimumab exhibit slow absorption and distribution. Consequently, they are not preferred treatment options during the induction period (Sandborn et al., 2012). Our findings also support the observation in the recent head-to-head VARSITY (Sands et al., 2019b) trial that vedolizumab is more effective than adalimumab for long-term maintenance of clinical remission and endoscopic improvement. Vedolizumab selectively blocks the binding of α4β7 integrin to MAdCAM-1, thereby inhibiting the further migration of lymphocytes into the lamina propria and intestinal lymphoid tissue without blocking the inflammatory active lymphocytes (Li et al., 2018). Therefore, it shows poor efficacy in the induction phase. Once effective, it shows a sustained anti-inflammatory response. Moreover, vedolizumab is highly safe owing to its unique mechanism of action and its excellent ability to maintain clinical remission and endoscopic mucosal improvement makes it highly competitive for application in patients with primary and secondary non-responsive, or refractory UC than infliximab.

The formation of anti-drug antibodies leads to a reduction in serum concentrations of infliximab, which is a major reason for primary or secondary non-response in some patients. In patients with Crohn’s disease or rheumatoid arthritis, the combination of immunomodulators and infliximab had been observed to reduce the production of anti-drug antibodies and increase serum trough levels of infliximab (Klotz et al., 2007; Ruffolo et al., 2010). Our study also confirms that the combination of AZA and infliximab improves efficacy in UC patients, although the reasons for this change remain unclear. Perhaps the most informative results from our analysis is the first confirmation of the dominance of JAK inhibitors in treating moderate to severe UC. This is increasingly relevant given the high rates of primary nonresponse or secondary loss of response to initial biologic therapy, and is an often-faced clinical scenario for which there is limited guidance. We observed that upadacitinib was significantly more effective than other biologic therapies for induction and maintenance. Upadacitinib is a small molecule drug characterized by non-immunogenicity and high bioavailability. At the same time, upadacitinib is more selective than tofacitinib, exhibiting a greater inhibitory effect on JAK1 than on JAK2 and JAK3. This selectivity reduces some adverse events associated with JAK2 and JAK3 inhibition while improving efficacy (Napolitano et al., 2022). Findings from these indirect comparisons need to be interpreted with caution because these trials did not always mirror clinical practice. We acknowledge that there is a paucity of head-to-head trials to truly inform comparative efficacy and safety. However, it is important to note that across trials of therapy, key inclusion/exclusion criteria, outcome definitions, and patient and clinical characteristics, co-interventions were comparable across trials, which facilitated this network meta-analysis.

Besides inherent limitations of individual trials, there were limitations to our analyses. Most of the included trials relied on local investigators for endoscopic reading of endoscopic disease activity for trial recruitment and outcome assessment, whereas trials of tofacitinib, upadacitinib and ozanimod included blinded central readers, which can influence absolute event rates. There were differences in timing of outcomes assessment in induction and maintenance,and time-dependent variability in efficacy could not be analyzed in detail. At the same time, the follow-up times for AEs and SAEs are also somewhat inconsistent, which may potentially bias safety results.

Integrating findings from this meta-analysis and other studies, current evidence favors upadacitinib or infliximab as the preferred agents for induction and upadacitinib or vedolizumab as the preferred agents for maintenance in patients with moderate to severe UC. However, in addition to the quality of evidence, several other factors are important for facilitating shared decision-making and developing a personalized treatment strategy for each patient. These factors include the balance of the risk–benefit profile, specific patient attributes, the clinical judgment and experience of the treating physicians, patients’ values and preferences, as well as the costs and resources available. These considerations also shape healthcare policy regarding the positioning of different agents. Pragmatic head-to-head trials are warranted to optimally inform the relative positioning of newly available agents in clinical practice.

## Conclusion

In a systematic review and network meta-analysis, we found upadacitinib showed the best efficacy and safety in to be ranked highest in patients with moderate to severe ulcerative colitis. More trials of direct comparisons are needed to inform clinical decision making with greater confidence.

## Data Availability

The original contributions presented in the study are included in the article/Supplementary Material, further inquiries can be directed to the corresponding author.
