# Supplementary material for: Network meta-analysis of efficacy and safety of drugs for the treatment of moderate to severe ulcerative colitis
Source: Front Pharmacol. 2025 Jan 3;15:1481678. doi: 10.3389/fphar.2024.1481678 (PMC11739108; doi:10.3389/fphar.2024.1481678)
Supplement: Supplementary file 1 [file Table1.docx]

| Table S1A. Network meta-analysis of clinical remission rate during induction | | | | | | | | |
| --- | --- | --- | --- | --- | --- | --- | --- | --- |
| Infliximab |  |  |  |  |  |  |  |  |
| **2.70**  **(1.54,4.71)** | Adalimumab |  |  |  |  |  |  |  |
| **2.01**  **(1.12,3.60)** | 0.74 (0.55,1.00) | Vedolizumab |  |  |  |  |  |  |
| 1.44  (0.70,2.98) | 0.53 (0.27,1.04) | 0.72 (0.36,1.43) | Ustekinumab |  |  |  |  |  |
| 1.47  (0.70,3.10) | 0.55 (0.27,1.09) | 0.73 (0.36,1.49) | 1.02 (0.45,2.34) | Golimumab |  |  |  |  |
| 1.46  (0.71,3.02) | 0.54 (0.28,1.05) | 0.73 (0.37,1.45) | 1.02 (0.45,2.28) | 0.99 (0.43,2.28) | Tofacitinib |  |  |  |
| 0.50  (0.24,1.02) | **0.18 (0.10,0.36)** | **0.25 (0.13,0.49)** | **0.35 (0.16,0.77)** | **0.34 (0.15,0.77)** | **0.34 (0.15,0.76)** | Upadacitinib |  |  |
| 1.33  (0.63,2.84) | 0.49 (0.25,1.00) | 0.66 (0.32,1.36) | 0.93 (0.40,2.14) | 0.91 (0.39,2.13) | 0.91 (0.39,2.11) | **2.68 (1.17,6.14)** | Ozanimod |  |
| **4.70**  **(3.02,7.34)** | **1.74 (1.24,2.45)** | **2.34 (1.61,3.42)** | **3.27 (1.84,5.80)** | **3.20 (1.75,5.82)** | **3.21 (1.81,5.69)** | **9.43 (5.38,16.54)** | **3.52 (1.91,6.50)** | Placebo |

| Table S1B. Network meta-analysis of endoscopic improvement rate during induction | | | | | | | | | |
| --- | --- | --- | --- | --- | --- | --- | --- | --- | --- |
| Infliximab |  |  |  |  |  |  |  |  |  |
| 0.74 (0.39,1.39) | Infliximab/  AZA |  |  |  |  |  |  |  |  |
| **2.31 (1.49,3.57)** | **3.13 (1.45,6.74)** | Adalimumab |  |  |  |  |  |  |  |
| **1.96 (1.19,3.22)** | **2.66 (1.19,5.94)** | 0.85 (0.55,1.32) | Vedolizumab |  |  |  |  |  |  |
| 1.48 (0.87,2.51) | 2.00 (0.88,4.57) | 0.64 (0.40,1.04) | 0.75 (0.44,1.29) | Ustekinumab |  |  |  |  |  |
| **1.87 (1.13,3.10)** | **2.53 (1.13,5.69)** | 0.81 (0.51,1.27) | 0.95 (0.57,1.59) | 1.26 (0.73,2.18) | Golimumab |  |  |  |  |
| 1.27 (0.75,2.16) | 1.72 (0.75,3.93) | **0.55 (0.34,0.89)** | 0.65 (0.38,1.11) | 0.86 (0.49,1.52) | 0.68 (0.40,1.17) | Tofacitinib |  |  |  |
| **0.41 (0.24,0.72)** | 0.56 (0.24,1.30) | **0.18 (0.11,0.30)** | **0.21 (0.12,0.37)** | **0.28 (0.15,0.51)** | **0.22 (0.13,0.39)** | **0.33 (0.18,0.59)** | Upadacitinib |  |  |
| 1.19 (0.67,2.14) | 1.62 (0.68,3.82) | **0.52 (0.30,0.89)** | 0.61 (0.34,1.10) | 0.81 (0.44,1.50) | 0.64 (0.35,1.16) | 0.94 (0.51,1.74) | **2.88 (1.52,5.46)** | Ozanimod |  |
| **3.42 (2.42,4.83)** | **4.63 (2.25,9.53)** | **1.48 (1.14,1.93)** | **1.74 (1.22,2.49)** | **2.31 (1.55,3.46)** | **1.83 (1.27,2.64)** | **2.69 (1.80,4.01)** | **8.25 (5.33,12.77)** | **2.87**  **(1.79,4.57)** | Placebo |

| Table S1C. Network meta-analysis of clinical remission rate during maintenance | | | | | | | | | | | |  |
| --- | --- | --- | --- | --- | --- | --- | --- | --- | --- | --- | --- | --- |
| Infliximab |  |  |  |  |  |  | |  | |  | | |
| 1.13  (0.61,2.10) | Adalimumab |  |  |  |  |  | |  | |  | | |
| 0.74  (0.40,1.37) | **0.66**  **(0.49,0.88)** | Vedolizumab |  |  |  |  | |  | |  | | |
| 1.15  (0.58,2.29) | 1.02  (0.57,1.82) | 1.56  (0.88,2.76) | Ustekinumab |  |  |  | |  | |  | | |
| 1.29  (0.61,2.75) | 1.15  (0.59,2.22) | 1.74  (0.90,3.36) | 1.12  (0.54,2.30) | Golimumab |  |  | |  | |  | | |
| 0.68  (0.32,1.42) | 0.60  (0.32,1.14) | 0.92  (0.49,1.73) | 0.59  (0.29,1.19) | 0.53  (0.24,1.14) | Tofacitinib |  | |  | |  | | |
| 0.53  (0.24,1.15) | **0.47**  **(0.23,0.93)** | 0.71  (0.36,1.41) | **0.46**  **(0.22,0.96)** | **0.41**  **(0.18,0.92)** | 0.78  (0.35,1.72) | Upadacitinib | |  | |  | | |
| 1.10  (0.56,2.14) | 0.98  (0.56,1.70) | 1.49  (0.86,2.58) | 0.95  (0.51,1.79) | 0.85  (0.42,1.72) | 1.62  (0.82,3.21) | **2.09**  **(1.01,4.33)** | | Ozanimod | |  | | |
| **2.84**  **(1.71,4.74)** | **2.52**  **(1.77,3.59)** | **3.84**  **(2.71,5.42)** | **2.46**  **(1.56,3.89)** | **2.20**  **(1.26,3.84)** | **4.18**  **(2.46,7.12)** | **5.39**  **(2.99,9.74)** | | **2.58**  **(1.68,3.96)** | | Placebo | | |
| Table S1D. Network meta-analysis of endoscopic improvement during maintenance | | | | | | | | | | | | |
| Infliximab |  |  |  |  |  | |  | |  | |  | |
| **1.84**  **(1.03,3.30)** | Adalimumab |  |  |  |  | |  | |  | |  | |
| 1.03  (0.57,1.86) | **0.56**  **(0.43,0.73)** | Vedolizumab |  |  |  | |  | |  | |  | |
| 1.47  (0.75,2.86) | 0.80  (0.47,1.36) | 1.43  (0.83,2.45) | Ustekinumab |  |  | |  | |  | |  | |
| 1.51  (0.78,2.95) | 0.82  (0.48,1.40) | 1.47  (0.86,2.53) | 1.03  (0.55,1.92) | Golimumab |  | |  | |  | |  | |
| 0.97  (0.48,1.98) | **0.53**  **(0.29,0.95)** | 0.95  (0.52,1.71) | 0.66  (0.34,1.29) | 0.64  (0.33,1.26) | Tofacitinib | |  | |  | |  | |
| 0.70  (0.33,1.48) | **0.38**  **(0.20,0.72)** | 0.68  (0.36,1.29) | **0.48**  **(0.24,0.97)** | **0.46**  **(0.23,0.94)** | 0.72  (0.34,1.53) | | Upadacitinib | |  | |  | |
| 1.64  (0.87,3.10) | 0.89  (0.54,1.46) | 1.60  (0.97,2.64) | 1.12  (0.62,2.02) | 1.09  (0.60,1.96) | 1.69  (0.89,3.20) | | **2.34**  **(1.18,4.62)** | | Ozanimod | |  | |
| **3.85**  **(2.33,6.34)** | **2.09**  **(1.55,2.81)** | **3.73**  **(2.73,5.10)** | **2.62**  **(1.68,4.07)** | **2.54**  **(1.63,3.95)** | **3.95**  **(2.39,6.53)** | | **5.47**  **(3.14,9.53)** | | **2.34**  **(1.58,3.46)** | | Placebo | |

| Table S1E. Network meta-analysis of AE rate | | | | | | | | | | | | | | | | | | |
| --- | --- | --- | --- | --- | --- | --- | --- | --- | --- | --- | --- | --- | --- | --- | --- | --- | --- | --- |
| Infliximab |  | |  | |  | |  | |  |  | |  | | |  | |  | |
| 0.83 (0.43,1.60) | Infliximab/  AZA | |  | |  | |  | |  |  | |  | | |  | |  | |
| 1.13 (0.61,2.09) | 1.35 (0.55,3.32) | | Adalimumab | |  | |  | |  |  | |  | | |  | |  | |
| 1.42 (0.73,2.76) | 1.71 (0.67,4.33) | | 1.26  (0.96,1.67) | | Vedolizumab | |  | |  |  | |  | | |  | |  | |
| 1.30 (0.61,2.77) | 1.55 (0.57,4.23) | | 1.15  (0.65,2.02) | | 0.91  (0.49,1.68) | | Ustekinumab | |  |  | |  | | |  | |  | |
| 0.75 (0.36,1.56) | 0.90 (0.33,2.40) | | 0.66  (0.39,1.13) | | **0.52**  **(0.29,0.94)** | | 0.58 (0.29,1.15) | | Golimumab |  | |  | | |  | |  | |
| 1.38 (0.67,2.84) | 1.66 (0.63,4.39) | | 1.22  (0.73,2.04) | | 0.97  (0.55,1.71) | | 1.07 (0.54,2.10) | | 1.85 (0.96,3.55) | Tofacitinib | |  | | |  | |  | |
| 1.06 (0.49,2.32) | 1.28 (0.46,3.53) | | 0.94  (0.52,1.71) | | 0.75  (0.39,1.42) | | 0.82 (0.39,1.72) | | 1.42 (0.69,2.92) | 0.77 (0.38,1.55) | | Upadacitinib | | |  | |  | |
| 0.70 (0.36,1.39) | 0.85 (0.33,2.17) | | **0.63**  **(0.40,0.98)** | | **0.49**  **(0.30,0.82)** | | 0.54 (0.29,1.02) | | 0.94 (0.52,1.73) | **0.51 (0.28,0.91)** | | 0.66 (0.34,1.28) | | | Ozanimod | |  | |
| 1.18 (0.67,2.08) | 1.42 (0.60,3.36) | | 1.05  (0.82,1.34) | | 0.83  (0.59,1.17) | | 0.91 (0.55,1.51) | | 1.58 (0.98,2.54) | 0.86 (0.55,1.34) | | 1.11 (0.65,1.90) | | | **1.68 (1.15,2.44)** | | Placebo | |
| Table S1F. Network meta-analysis of SAE rate | | | | | | | | | | | | | | | | | |  |
| Infliximab | |  | |  | |  | |  |  | |  | |  |  | |  | |  |
| 0.14 (0.01,2.78) | | Infliximab/  AZA | |  | |  | |  |  | |  | |  |  | |  | |  |
| 0.87 (0.45,1.68) | | 6.20 (0.29,130.88) | | Adalimumab | |  | |  |  | |  | |  |  | |  | |  |
| 0.98 (0.51,1.87) | | 6.95 (0.33,146.67) | | 1.12 (0.79,1.59) | | Vedolizumab | |  |  | |  | |  |  | |  | |  |
| 0.91 (0.36,2.27) | | 6.44 (0.28,145.42) | | 1.04 (0.46,2.32) | | 0.93 (0.42,2.06) | | Ustekinumab |  | |  | |  |  | |  | |  |
| 0.49 (0.20,1.20) | | 3.46 (0.15,77.82) | | 0.56 (0.26,1.22) | | 0.50 (0.23,1.09) | | 0.54 (0.20,1.48) | Golimumab | |  | |  |  | |  | |  |
| 1.04 (0.38,2.87) | | 7.36 (0.32,171.48) | | 1.19 (0.48,2.97) | | 1.06 (0.43,2.64) | | 1.14 (0.37,3.50) | 2.13 (0.71,6.41) | | Tofacitinib | |  |  | |  | |  |
| 1.59 (0.60,4.21) | | 11.24 (0.49,258.61) | | 1.81 (0.76,4.34) | | 1.62 (0.68,3.86) | | 1.75 (0.59,5.16) | 3.25 (1.12,9.44) | | 1.53 (0.47,4.91) | | Upadacitinib |  | |  | |  |
| 1.23 (0.48,3.14) | | 8.72 (0.38,198.25) | | 1.41 (0.61,3.22) | | 1.25 (0.55,2.86) | | 1.35 (0.47,3.87) | 2.52 (0.90,7.07) | | 1.18 (0.38,3.69) | | 0.78 (0.26,2.33) | Ozanimod | |  | |  |
| 0.79 (0.45,1.37) | | 5.57 (0.27,115.51) | | 0.90 (0.64,1.27) | | 0.80 (0.57,1.12) | | 0.87 (0.42,1.79) | 1.61 (0.80,3.26) | | 0.76 (0.32,1.77) | | 0.50 (0.22,1.11) | 0.64 (0.30,1.36) | | Placebo | |  |

Comparisons of table S1A to S1F should be read from left to right. Numbers in bold are statistically significant. Numbers in parentheses indicate 95% CI.
