# Supplementary figures and images for: Network meta-analysis of efficacy and safety of drugs for the treatment of moderate to severe ulcerative colitis
Source: Front Pharmacol. 2025 Jan 3;15:1481678. doi: 10.3389/fphar.2024.1481678 (PMC11739108; doi:10.3389/fphar.2024.1481678)

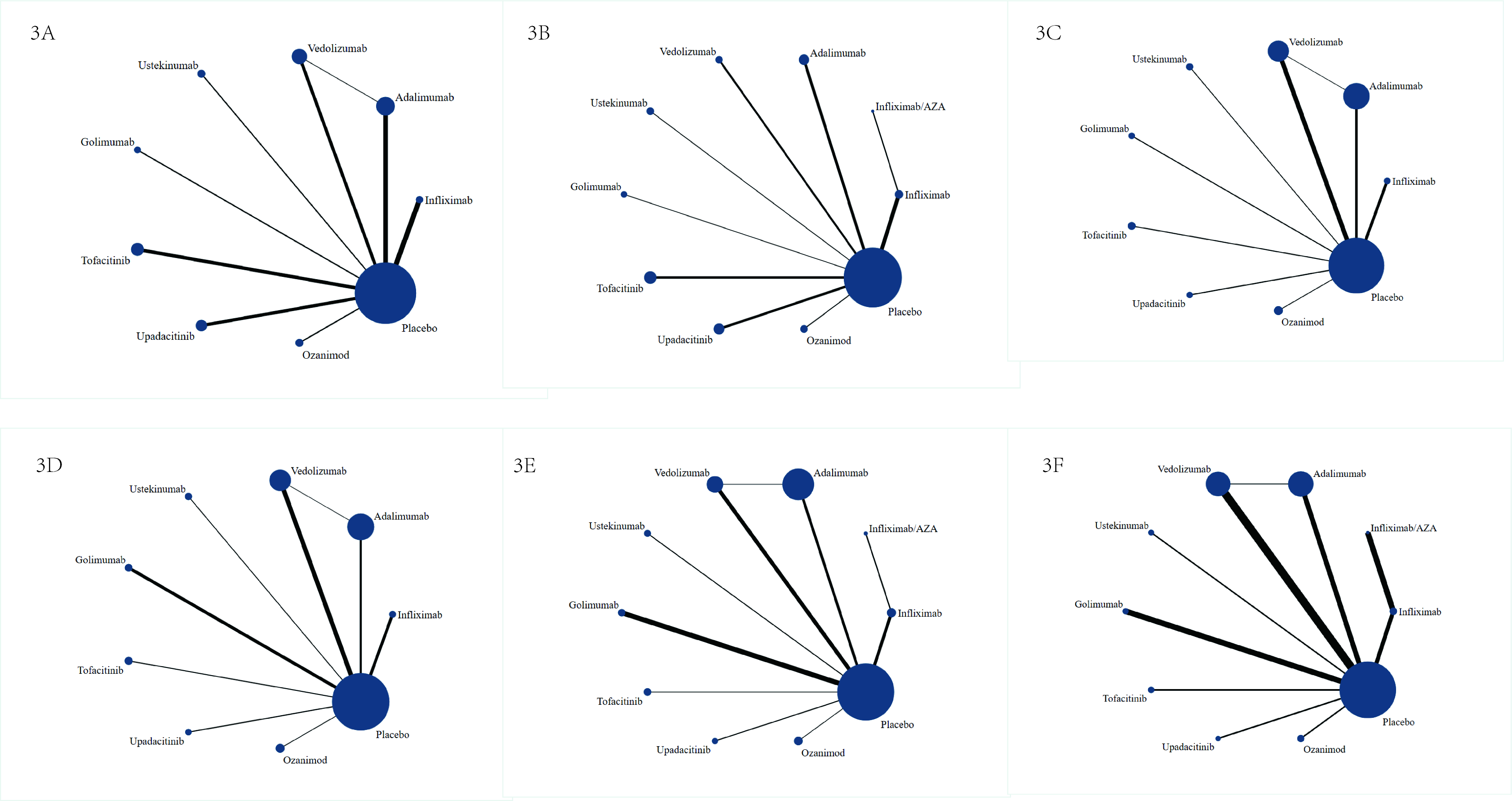

Supplement: Supplementary file 2 [file Image3.tif]

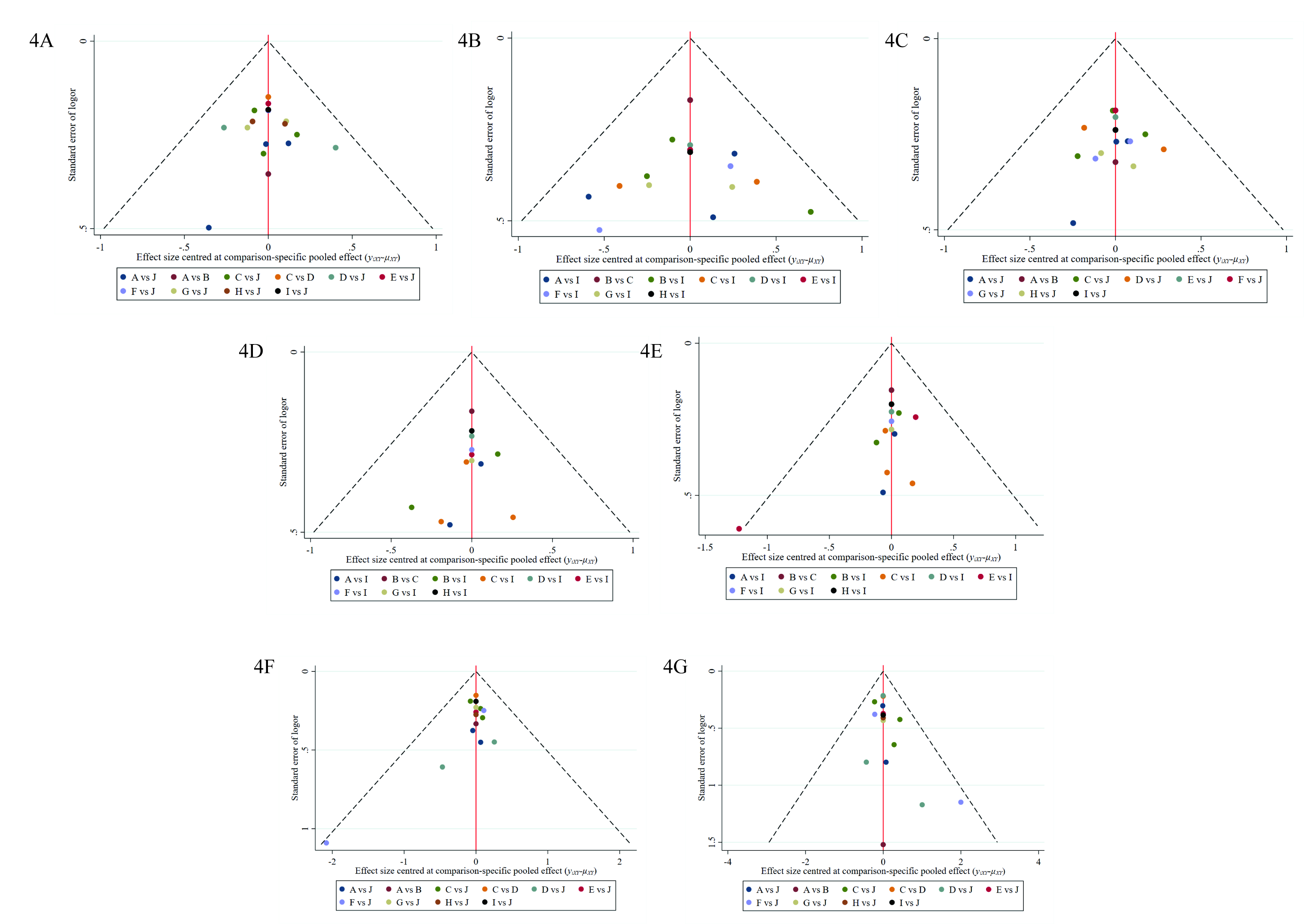

Supplement: Supplementary file 3 [file Image4.tif]

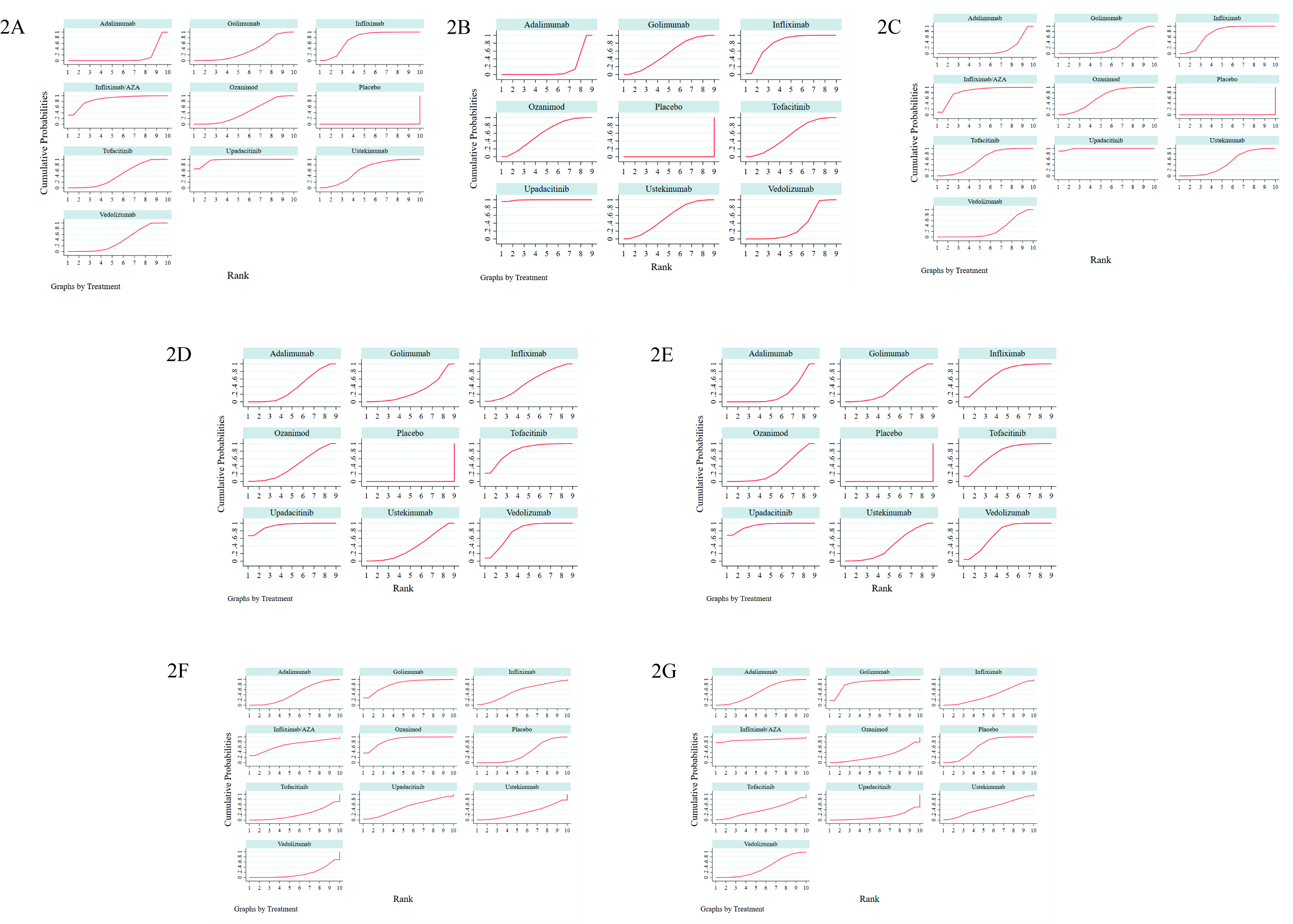

Supplement: Supplementary file 4 [file Image2.tif]

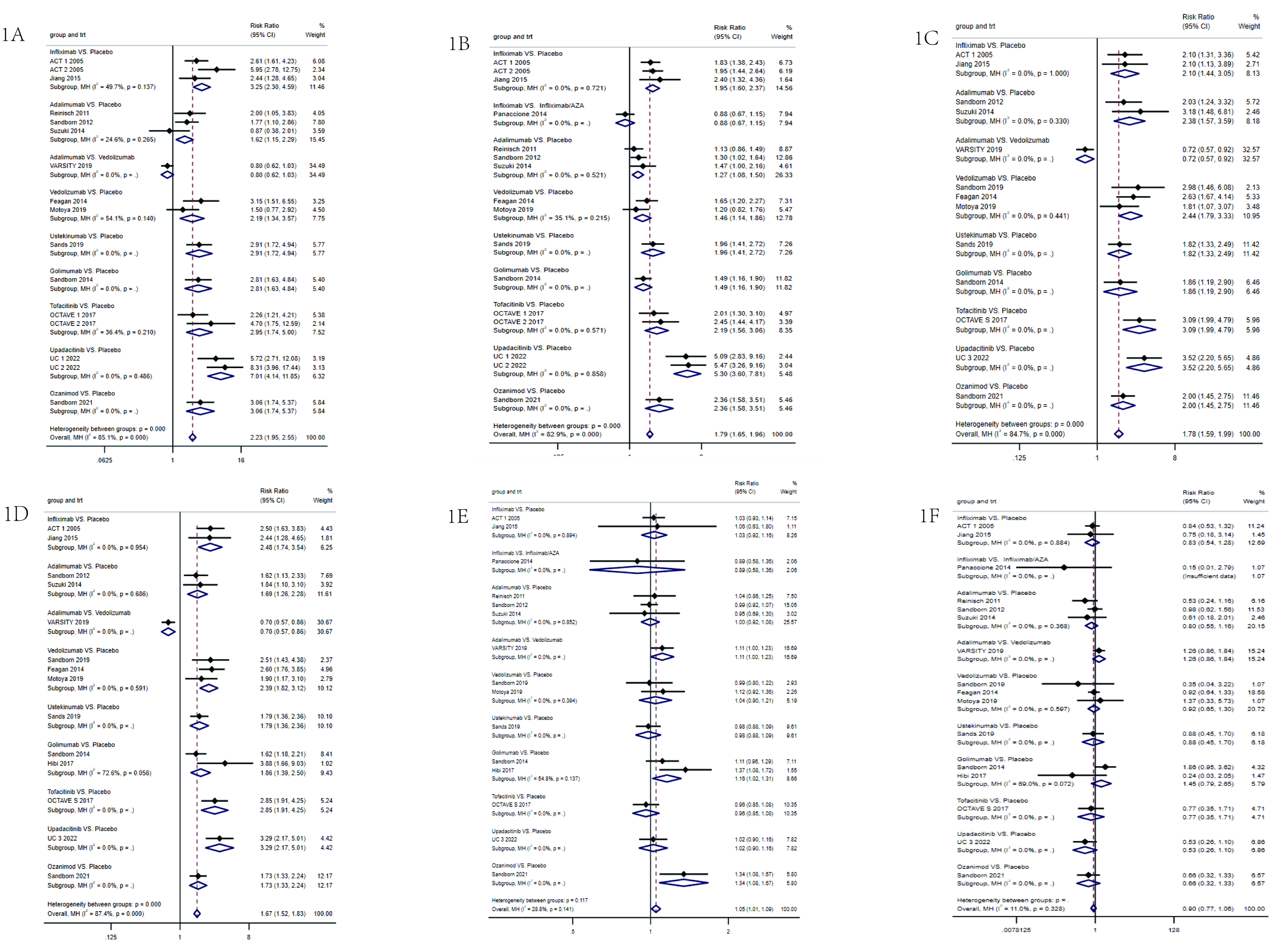

Supplement: Supplementary file 5 [file Image1.tif]

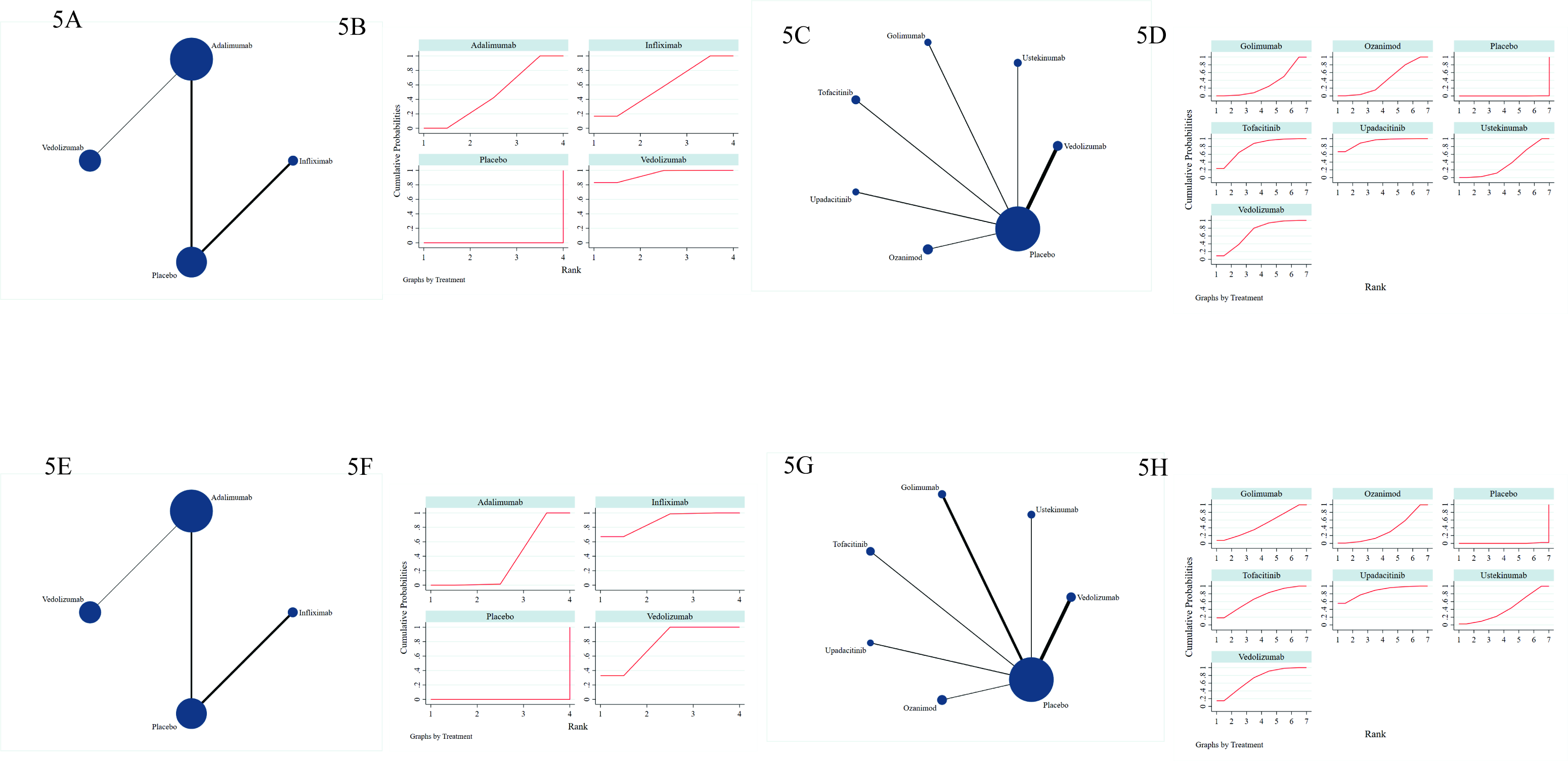

Supplement: Supplementary file 6 [file Image5.tif]
